# Effects of the Allelopathic Macroalga *Galaxaura divaricata* on the Foraging Behaviors of Reef Fishes Across Herbivorous and Nonherbivorous Feeding Guilds

**DOI:** 10.1002/ece3.73106

**Published:** 2026-02-17

**Authors:** Chen‐Lu Lee, Pi‐Jen Liu, Shao‐Lun Liu, Hsing‐Juh Lin

**Affiliations:** ^1^ Department of Biology National Museum of Natural Science Taichung Taiwan; ^2^ Graduate Institute of Marine Biology National Dong Hwa University Checheng Pingtung Taiwan; ^3^ Department of Life Science & Center for Ecology and Environment Tunghai University Taichung Taiwan; ^4^ Department of Life Sciences and Innovation and Development Center of Sustainable Agriculture National Chung Hsing University Taichung Taiwan

**Keywords:** allelopathy, coral reef fishes, Dongsha atoll, *Galaxaura divaricata*, herbivory

## Abstract

Macroalgal overgrowth‐induced phase shifts pose a significant threat to coral reef ecosystems. While herbivores can help control macroalgae biomass in reefs, some allelopathic macroalgae can resist herbivores from foraging. This resistance can lead to a decrease in herbivory levels. However, the impact of these allelopathic effects on other feeding groups of reef fish remains unclear. We examined the presence of the allelopathic macroalga: *Galaxaura divaricata* on the foraging behaviors of various reef fish species from different feeding guilds, including ten herbivorous and ten nonherbivorous species, in designed tank experiments. Most herbivorous fishes exhibited reductions of more than 60% in feeding on palatable macroalgae when exposed to *Galaxaura*. Nevertheless, the extent of feeding reduction differed among herbivorous species and feeding guilds, suggesting species‐specific variation in tolerance. *Galaxaura* had varying effects on the feeding quantity of shrimp among different species of nonherbivorous fish. The presence of *Galaxaura* did not significantly affect the feeding quantity of most Labridae, Balistidae, and Pomacanthidae fishes, but did physically reduce the feeding quantity of some large carnivorous fishes (e.g., Haemulidae, Lutjanidae, and Serranidae). There was a high variation in the effect on the feeding quantity of opportunistic corallivorous fishes. Our results indicate that allelopathic macroalgal dominance can influence not only herbivorous fishes that regulate algal biomass, but also nonherbivorous fishes. Such changes in foraging may alter trophic interactions in coral reef ecosystems. This also suggests that maintaining functional diversity in fish feeding guilds and preserving specific herbivorous functions are crucial for the resilience of coral reefs against the phase shift caused by macroalgal overgrowths.

## Graphical abstract

Herbivorous fishes reduce their feeding with allelopathic macroalga: Galaxaura, while nonherbivorous fishes didn't change their feeding with Galaxaura. Feeding reduction with Galaxaura differed across fish species and may vary by morphology or chemical resistance of different fish species.

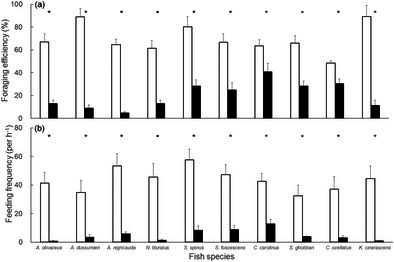

## 1 Introduction

Anthropogenic disturbances, such as eutrophication and overfishing, have contributed to the overgrowth of macroalgae and the decline of corals, leading to a “phase shift” from coral‐dominated to macroalgae‐dominant systems (Hughes et al. 2007; Liu et al. 2009; Norström et al. 2009). To prevent or reverse this phase shift, marine herbivores have been proposed as a solution to effectively remove large amounts of macroalgal biomass and restore coral cover in degraded reef systems (Crisp et al. 2022; Hughes et al. 2010). Herbivores comprise multiple functional groups in the reef (e.g., grazers, browsers and scrapers). Their complementary functional roles can effectively suppress the overgrowth of macroalgae in benthic environments (Choat et al. 2002; Hoey and Bellwood 2009). Although the regulation of herbivores on algal biomass can prevent macroalgae overgrowth, some macroalgae have evolved defensive mechanisms, such as the production of rich secondary metabolic chemicals, to reduce their palatability and defend against herbivory (Duffy and Hay 1994; Rasher and Hay 2010; Semmouri et al. 2024). In turn, herbivory avoidance may allow these chemical‐rich macroalgae to outcompete corals during macroalgae overgrowth, negatively impacting coral survival and recovery (Fong et al. 2019; Rasher and Hay 2010; Smith et al. 2006). Nevertheless, the strong repellent characteristics of chemically rich macroalgae can also allow them to serve as a refuge for neighboring food sources or prey (i.e., other palatable macroalgae or benthic invertebrates), which may eventually alter the trophic pathways in coral reef systems (Brooker et al. 2016, 2017; McCormick et al. 2017).

The overgrowth of allelopathic macroalgae has increasingly occurred in some degraded coral reefs in recent decades (Doropoulos et al. 2014, 2017; Nieder et al. 2019; Roff, Chollett, et al. 2015; Roff, Doropoulos, et al. 2015). Some herbivorous (Doropoulos et al. 2014, 2017; Loffler et al. 2015; Roff, Doropoulos, et al. 2015) and corallivorous (Brooker et al. 2016, 2017) fish may reduce or avoid feeding on their preferred food sources in the presence of nearby allelopathic macroalgae, likely due to physical and chemical interference. Although the impacts of allelopathic macroalgae on herbivorous fishes are increasingly recognized, the effects of allelopathic macroalgae on different feeding guilds of reef fishes remain unexplored. Macroalgal phase shifts can not only affect herbivory but also restructure reef habitats and modify prey availability, potentially influencing a wide range of fishes and altering trophic transfer pathways. Understanding these mechanisms would help assess the ecological impacts associated with coral reef phase shifts. This, in turn, raises several important questions: Do these chemical‐rich macroalgae affect all herbivorous fish in the same way? Do these macroalgae have the same repellent effects on carnivorous, omnivorous, and other nonherbivorous fishes? In addition, different fish species within the same feeding guild may have distinct foraging adaptations for allelopathic macroalgae (Brooker et al. 2016; Loffler et al. 2015; Puk et al. 2020; Rasher and Hay 2010). Such key information on the interactions between fish feeding guilds and allelopathic macroalgae is essential for a better understanding of the dynamics of reef fish communities concerning the resilience mechanisms of degraded coral reef ecosystems in response to increasing overgrowths of allelopathic macroalgae.

The red alga genus *Galaxaura* is characterized by both coarse calcareous thalli and rich secondary metabolite chemicals, which together confer physical and chemical defenses, making this macroalga unpalatable to most herbivorous fishes (Mantyka and Bellwood 2007; McCormick et al. 2017; Rasher et al. 2013). The red alga species, *Galaxaura divaricata*, has been shown to have overgrown and facilitated an obvious coral reef phase shift in the Dongsha Atoll, South China Sea (Nieder et al. 2019, 2022). Previous field studies have found that its presence may potentially influence the feeding behavior of coral reef fishes (Nieder et al. 2022) and other herbivorous animals, for example, sea urchins and green turtles (Nieder et al. 2024). However, there is currently insufficient quantitative information to demonstrate its effects on various coral reef fish species. The main goal of this study was to examine the effects of the allelopathic macroalga *G. divaricata* by (1) confirming its allelopathic effects on benthic scleractinian corals, and (2) testing its influence on the foraging behaviors of reef fishes across different feeding guilds. We hypothesized that the presence of *G. divaricata* would exert allelopathic effects on corals. In addition, we expected that the foraging efficiency of reef fishes would decrease in the presence of *G. divaricata* , with the magnitude of this decrease varying among fish species and trophic guilds.

## 2 Materials and Methods

### 2.1 Study Organisms: Allelopathic Algae

We documented feeding interactions of coral reef fish with the allelopathic macroalga *Galaxaura divaricata* by a series of tank experiments in the National Museum of Marine Biology and Aquarium (NMMBA). Our previous studies have shown that this alga is highly allelopathic and repels feeding behaviors of many marine herbivorous animals (Nieder et al. 2019, 2024). Here we collected *Galaxaura divaricata* (hereafter referred to as *Galaxaura*) from Dongsha Atoll, South China Sea, in October 2016. All fresh *Galaxaura* thalli were kept in flow‐through aquaria tanks (water temperature: 25°C–26°C, 12 h of light per day) in the NMMBA for at least 1 month before our experiments.

Although several *Galaxaura* species are known to exert strong allelopathic effects on corals, the allelopathic potential of *G. divaricata* remains unknown. To confirm the potential allelopathic capacity of *G. divaricata* , we conducted preliminary tank experiments from December 2016 to February 2017 to evaluate its effects on hard corals. Two hard coral species, *Pocillopora damicornis* and *Porites cylindrica*, were selected for this test because they are two of the dominant coral species in Dongsha Atoll. Branches of 7–10 cm in length of *P. damicornis* and *P. cylindrica* were selected and fixed onto 10 × 10 cm tiles by water‐proofing glue in a 2000‐L flow‐through tank (water temperature: 25°C–26°C, 12 h of light per day). Three treatments (ten replicates in each coral species for each treatment) were implemented to test the negative effects of *Galaxaura* on corals (modified from Del Monaco et al. 2017; Rasher and Hay 2010). (1) Control treatment: coral branch only, without any manipulation; (2) *Galaxaura* treatment: *Galaxaura* fragments (fresh weight of 2 g, approximately 3 to 5 cm‐long fragments) were fixed on coral branch with plastic cable‐tie, positioned 1–2 cm from the tip of the branch.; and (3) Mimic treatment: To distinguish the physically contact or shading effect of *Galaxaura* in the preliminary test, we fixed a *Galaxaura*‐like plastic seaweed with similar shape and color as a dummy (approximately 3–5 cm in length, similar in size to the *Galaxaura* treatments) to the coral branch with plastic cable‐tie, 1–2 cm from the tip of the branch. A total of 27 replicates (9 individual branches × 3 treatments) were conducted for each coral species. After 14 days, all coral branches were retreated and photographed. The bleaching area of each branch was assessed via Image‐Pro Plus software and expressed as a percentage (%) relative to the total area of the coral branch.

### 2.2 Study Organisms: Fish and Their Target Food

Ten species of herbivorous fishes ( *Acanthurus dussumieri* , *Acanthurus nigricauda* , *Acanthurus olivaceus* , *Naso lituratus* , *Siganus fuscescens* , *Siganus spinus* , *Calotomus carolinus* , *Cetoscarus ocellatus*, *Scarus ghobban* , and *Kyphosus cinerascens* ) and ten species of nonherbivorous fishes ( *Epinephelus merra* , *Lutjanus fulviflamma* , *Plectorhinchus chaetodonoides* , *Rhinecanthus aculeatus* , *Coris aygula* , *Halichoeres nebulosus* , *Chaetodon auripes* , *Chaetodon lunula* , *Chaetodon vagabundus* , and *Pomacanthus semicirculatus* ) were selected to represent different feeding guilds (Froese and Pauly 2016) in this study (Table 1). These fish were all common reef fish species in the Dongsha coral reefs. Before the main experiments, fishes were collected in the field and maintained in separate flow‐through aquaria of appropriate size and acclimated by species for at least 1 week to allow for acclimation at NMMBA. After 1 week of acclimation, any fish that exhibited signs of stress—such as weakness or inappetence—were excluded from the subsequent experiments. Finally, six individuals of each fish species were used in the following experiments. Fish handling and experimental procedures followed the institutional guidelines for animal care and welfare at NMMBA. No invasive or lethal procedures were applied during our study.

**TABLE 1:** Twenty tested fish species, their total body length range, and feeding guilds in this study. Each trial included six individuals of each fish species. The feeding guild classifications of the 20 fish species were based on information from FishBase.

Family	Species	Total body length range (cm)	Feeding guild
**Herbivorous fishes**			
Acanthuridae	*Acanthurus olivaceus*	12 ~ 15	Browser
	*Acanthurus dussumieri*	18 ~ 30	Browser
	*Acanthurus nigricauda*	15 ~ 25	Browser
	*Naso lituratus*	19 ~ 28	Browser
Siganidae	*Siganus spinus*	8 ~ 12	Grazer
	*Siganus fuscescens*	10 ~ 16	Grazer
Scaridae	*Calotomus carolinus*	14 ~ 19	Browser
	*Scarus ghobban*	15 ~ 21	Scraper
	*Cetoscarus ocellatus*	16 ~ 19	Scraper
Kyphosidae	*Kyphosus cinerascens*	24 ~ 30	Browser
**Nonherbivorous fishes**			
Balistidae	*Rhinecanthus aculeatus*	9 ~ 11	Carnivores
Haemulidae	*Plectorhinchus chaetodonoides*	14 ~ 16	Carnivores
Lutjanidae	*Lutjanus fulviflamma*	9 ~ 17	Carnivores
Serranidae	*Epinephelus merra*	13 ~ 18	Carnivores
Labridae	*Coris aygula*	12 ~ 19	Carnivores
	*Halichoeres nebulosus*	10 ~ 17	Carnivores
Chaetodontidae	*Chaetodon auripes*	15 ~ 25	Opportunistic coral feeders
	*Chaetodon lunula*	16 ~ 25	Opportunistic coral feeders
	*Chaetodon vagabundus*	15 ~ 22	Opportunistic coral feeders
Pomacanthidae	*Pomacanthus semicirculatus*	24 ~ 32	Omnivores

Fresh brown alga, *Dictyota bartayresiana* (hereafter referred to as *Dictyota*), was represented as target food for herbivorous fish. This macroalgae often coexist with *Galaxaura* in the same reef habitat at Dongsha. We collected thalli of *Dictyota* from Dongsha and cultivated them in flow‐through tanks at NMMBA. In the case of nonherbivorous fish, fresh shrimp meat (*Penaeus* spp., hereafter referred to as shrimp) was selected as the target food source instead of live crustaceans to minimize experimental variability caused by unpredictable prey movement. All fish were maintained in tanks (without *G. divaricata* ) and were fed the designated food items. The *Dictyota* and shrimp were fed daily for herbivorous fish species and nonherbivorous fish species, respectively, during the acclimation periods. Most fish consumed approximately 100% of their target food, confirming their acceptance of *Dictyota* or shrimp as ideal food sources during the acclimation period. Only individuals that consistently consumed the provided food were used in subsequent experiments.

### 2.3 Experimental Design

**FIGURE 1:**
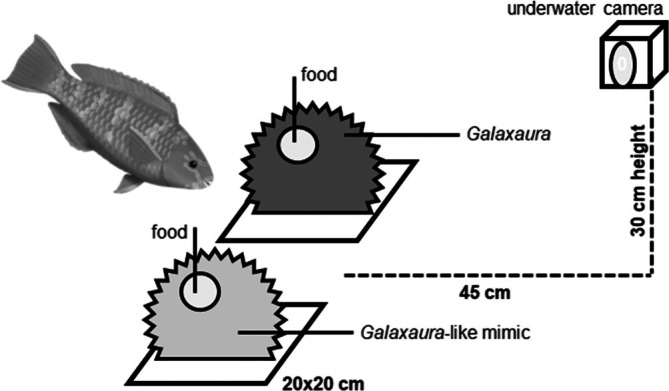
Tank design of our main experiment. In each trial, one fish was presented with two separated bowls with the designated food inside.

Our main experiments were conducted from February to March 2017. A two‐sided choice cafeteria‐style experiment was designed to test the effect of chemical‐rich algae on feeding preferences. All experimental trials were carried out in a series of 120 × 75 × 75 cm flow‐through tanks. In the experimental tanks, water circulation was maintained using a 250‐W pump, ensuring that the entire tank volume was circulated at least once per hour. Filtered seawater was sourced from NMMBA, with a daily exchange rate of approximately 10%–20%. The tanks were illuminated by two 400‐W HQI (Hydrargyrum quartz iodide) metal halide lamps, which delivered an irradiance of 300–400 μmol m^−2^ s^−1^ in photosynthetically active radiation (PAR) with a 12‐h light cycle each day. All experiments took place during this light period. Salinity was maintained at 33–34 psu, and the water temperature was kept between 25°C and 26°C. The water temperature was consistent with the spring temperature of the Dongsha Atoll. In each tank, two 20 × 20 × 3 cm plastic bowls containing the designated food were placed 30 cm apart (Figure 1). Two experimental designs were set to assess the effects of *Galaxaura* on the foraging behaviors of different reef fish species. For each fish species, six individuals (of similar body length) were used as replicates in the experimental trials. Each fish individual participated in the experimental trial only once.

In our main experiment, we aimed to test the presence of allelopathic macroalgae on the foraging behaviors of reef fishes (Figure 1a). In this study, we emphasized simulating the foraging behavior of fish in response to algae canopies in natural habitats. Therefore, we used fresh *Galaxaura* thallus that retained its natural structure with a dense, corymbose branching morphology, along with visually similar plastic mimics for testing, rather than using extracted chemical compounds from *Galaxaura*. In each trial, a fully mature *Galaxaura* thallus and the plastic mimic were used as the treatments and placed in separate bowls within the experiment tank. The *Galaxaura* treatments consisted of a *Galaxaura* thallus approximately 10 cm in diameter (approximately fresh weight of 30 g), representing natural conditions commonly observed on reefs at Dongsha. The plastic mimic treatments consisted of a *Galaxaura*‐like plastic seaweed with a similar size (approximately 10 cm in diameter) as the *Galaxaura* treatments. The treatments were placed in separate bowls inside the tank for each trial, with the position and orientation of the *Galaxaura* and plastic mimic bowls randomized. Food items for the herbivorous or nonherbivorous fish were then set in the bowls in the experiment tanks. For the herbivorous fish trials, 5 g (fresh weight) of *Dictyota* thallus was fixed on the *Galaxaura* and plastic mimic treatments using cable ties. For the nonherbivorous fish trials, six pieces of shrimp meat (each about 2 g in weight, without shell) were carefully inserted into the *Galaxaura* or plastic mimic treatments using forceps, positioned 2 cm beneath the surface of the *Galaxaura* or plastic mimic thallus.

In this tank experiment, an individual fish was free to select their food source from the *Galaxaura* or plastic mimic treatment using their senses. Before each experiment trial, the individual fish was rested and was starved in separate tanks for 24 h to ensure gut evacuation. A duration exceeding reported digestion times for various herbivorous and carnivorous reef fishes (Choat 1991; Choat and Bellwood 1985; Palomares and Pauly 1989). Each experimental trial involved only a single fish individual. At the beginning of each trial, the single fish individual was carefully transferred to the experiment tank in a mesh basket to acclimate to the water conditions for 30 min. During this period, they could receive visual and olfactory cues from the experiment tank but could not access the food (modified from Brooker et al. 2017). After 30 min, the fish was released into the experiment tank for a 1‐h testing trial. At the end of the trial, we retrieved the bowls and measured the weight loss of *Dictyota* (in the herbivorous fish trials) or the number of remaining shrimp meat (in the nonherbivorous fish trials), which represented the fish foraging efficiency. The loss of *Dictyota* was standardized using another caged *Dictyota* as the control, following the formula of Cronin and Hay 1996. All the *Galaxaura* thalli in each treatment were replaced before each trial. After each trial, we drained all seawater from the tank and removed any food debris, algal fragments, and fish feces in the tank to avoid possibly affecting the next set of experiments.

In addition to quantifying interactions of reef fishes with *Galaxaura* thalli and plastic‐mimic treatments, we deployed a BenQ SP2 underwater camera in our experiment tank during the main experiment. The camera was mounted on a weight at the tank bottom at a height of 30 cm, and was positioned 45 cm from the experiment bowls for an overview. We aimed to record the feeding frequencies of fish during each experiment over a duration of 1 h. The first and last 3 min of the recorded films were omitted from the analysis to allow settling time after camera deployment and to avoid disturbance caused by the manipulations. The feeding frequency count was referred to in our previous study (Nieder et al. 2022). An individual bite number was counted when the fish directly contacted *Galaxaura* with its mouth, with the following biting (by herbivores) or striking (by nonherbivores) behaviors of each fish. A “biting” and “strike” were defined as a rapid forward thrust of the snout or mouth toward the algal thallus, indicative of an attempted feeding action aimed at accessing food items placed within the macroalgal structure. Ultimately, we recorded the feeding frequency of individual fish on associated food in *Galaxaura* or plastic mimics during the experiment.

### 2.4 Statistical Analysis

The consumption percentage of *Dictyota* biomass (for herbivorous fishes) or shrimp numbers (for nonherbivorous fishes) was calculated to represent the foraging efficiency as a percentage (%). The bite or striking numbers counting was represented as the feeding frequency. For each fish species, the results of six individuals served as replicates in the tank experiment. All data sets were tested for homogeneity of variance using Levene's test before further statistical analysis. To evaluate whether tank identity or fish size influenced treatment responses, we additionally analyzed our data sets using linear mixed‐effects models (LMMs). In these models, treatment, tank, and species were included as fixed effects, fish standard length (TL) was included as a covariate, and replicate identity was included as a random intercept. Sensitivity analyses further included a treatment × tank interaction to test for potential container‐dependent treatment effects. Model selection was based on Akaike's Information Criterion (AIC). Across all experiments, tank identity did not significantly explain variation in foraging responses, and critically, the treatment × tank interaction was not significant (feeding efficiency: *p* = 0.199; feeding frequency: *p* = 0.594), indicating that tank identity effects did not alter treatment outcomes. Model comparison based on AIC further showed that including tank or its interaction with treatment did not improve model fit (ΔAIC < 2). Since most tank experimental trial data sets are related to the same tank unit, this violates the parametric assumption. A nonparametric Friedman test was used to test the coral bleaching area of three treatments (Control vs. *Galaxaura* vs. plastic mimic) in the preliminary test. Finally, the Wilcoxon signed‐rank tests were used to test the differences in feeding frequency (count numbers) as well as foraging efficiency (%) of different fish species in the tank experiments. All the statistics were analyzed by SPSS 16.

## 3 Results

### 3.1 Effect on Coral

**FIGURE 2:**
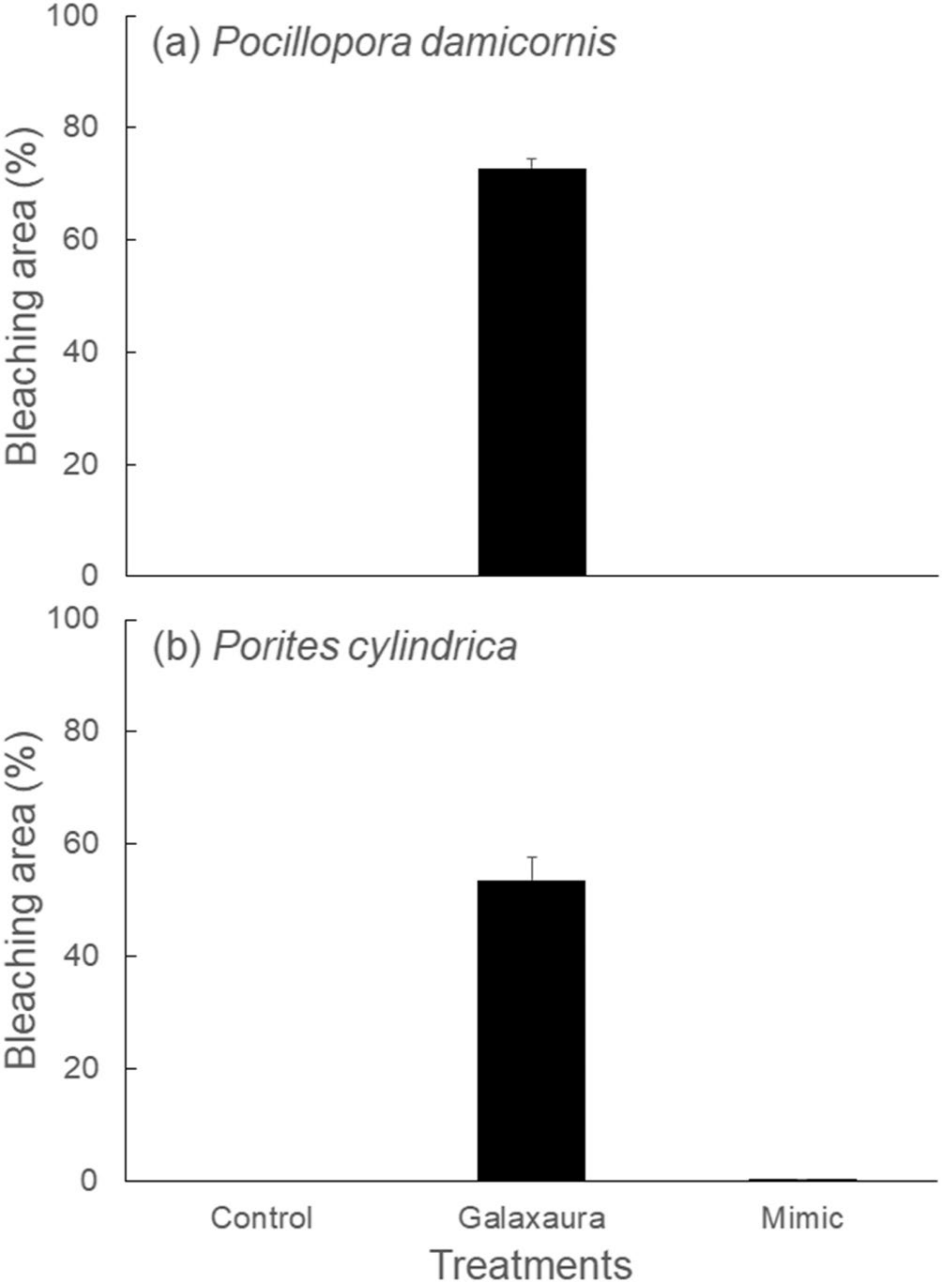
Coral bleaching area of (a) *Pocillopora damicornis* and (b) *Porites cylindrica* in the three treatments of the preliminary test (mean ± SE).

Our preliminary test indicated that *Galaxaura* could suppress hard corals (Figure 2). Noticeably bleaching areas were observed on both *Pocillopora damicornis* (72.83% ± 1.55%) and *Porites cylindrica* (53.58% ± 3.97%) with the *Galaxaura* treatments. The bleaching areas in the *Galaxaura* treatments for two coral species were significantly higher than those in the control and plastic mimic treatments (Friedman test: *p* < 0.001), indicating that the negative effects on corals were not caused by physical shading or physical contact with *Galaxaura*.

### 3.2 Effect on Herbivorous Fish Species

**FIGURE 3:**
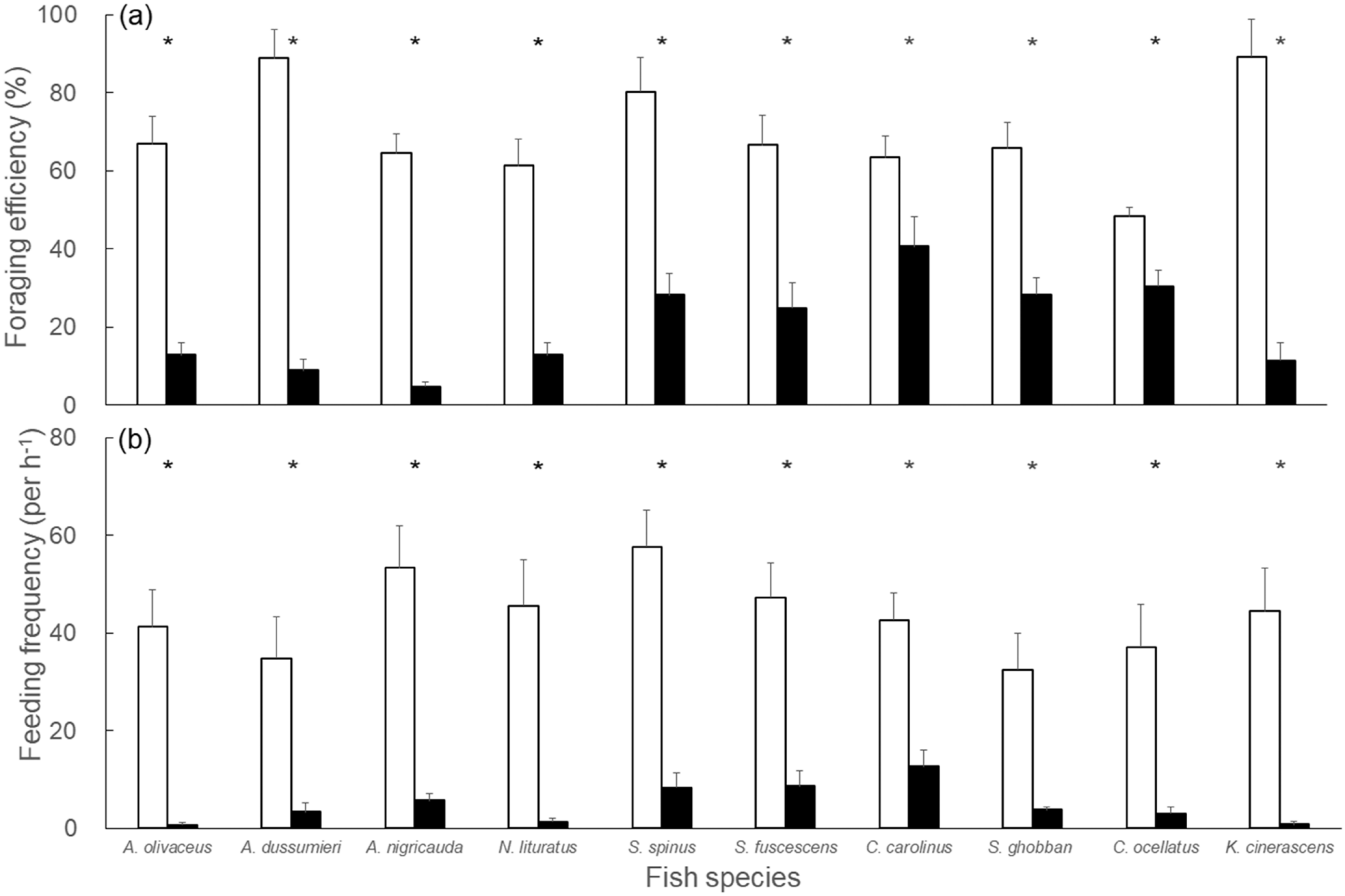
(a) Feeding efficiency and (b) feeding frequency of ten herbivorous fish species (mean ± SE). White: Plastic mimic treatment; black: *Galaxaura* treatment. Asterisks represent significant differences in statistics between the two treatments.

For most herbivorous fish species, their foraging efficiencies (Figure 3a) and feeding frequencies (Figure 3b) were significantly lower in the treatments with *Galaxaura* across all experiments (Wilcoxon signed‐rank tests: *p* < 0.05). Four species of acanthurids and *Kyphosus cinerascens* exhibited strong repellent behaviors toward the food in the *Galaxaura* treatments in the tank experiment. Algal foraging efficiency for these five species was approximately 60%–90% in the plastic mimic treatments, but it dramatically decreased to less than 10% in the *Galaxaura* treatments (Figure 3a). The foraging efficiencies of two siganids and three scarids also significantly decreased in the *Galaxaura* treatments. However, these fishes consumed more *Dictyota* biomass (approximately 30%–40%) than the acanthurids and *K. cinerascens* in the *Galaxaura* treatments. Among all herbivorous fish, the scarid species *Calotomus carolinus* demonstrated the highest foraging efficiencies in the *Galaxaura* treatments, consuming approximately 51.52% ± 5.08% of *Dictyota* biomass in *Galaxaura* treatments. In terms of behavior, the ten herbivorous fish species clearly preferred to approach and feed on food sources on plastic mimic treatments (Figure 3b). Although most herbivorous fish significantly avoided the *Galaxaura* treatments while foraging, two siganids and *C. carolinus* exhibited slightly higher feeding frequencies on *Galaxaura* treatments than other acanthurids and *K. cinerascens* .

### 3.3 Effect on Nonherbivorous Fish Species

**FIGURE 4:**
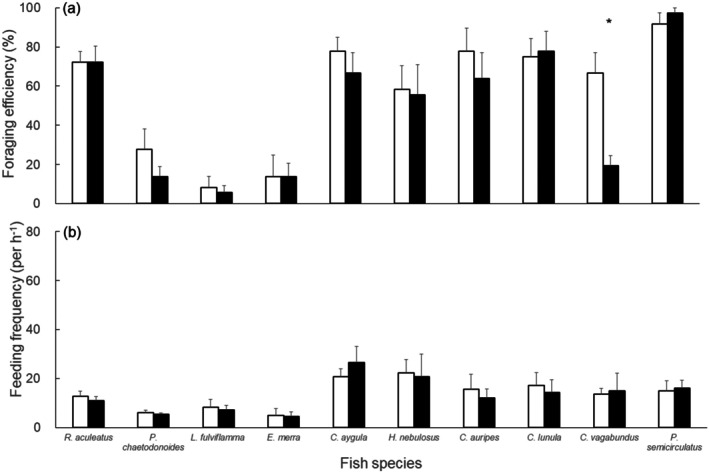
(a) Feeding efficiency and (b) feeding frequency of ten nonherbivorous fish species (mean ± SE). White: Plastic mimic treatment; black: *Galaxaura* treatment. Asterisks represent significant differences in statistics between the two treatments.

Foraging efficiency varied notably among nonherbivorous fish species. Most nonherbivorous fish species had no significant differences in foraging efficiencies (Figure 4a) and feeding frequencies (Figure 4b) between the *Galaxaura* and plastic mimic treatments in the tank experiment (Wilcoxon signed‐rank tests: *p* > 0.05). However, there were obvious differences in feeding efficiency among fish species. Three carnivorous fish species ( *Epinephelus merra* , *Lutjanus fulviflamma*, and *Plectorhinchus chaetodonoides* ) showed significantly lower foraging efficiencies compared to the other nonherbivorous species in both *Galaxaura* and plastic mimic treatments. These species consumed only a few shrimps across all the treatments, with foraging efficiency below 30%. In contrast, two labrid species ( *Coris aygula* and *Halichoeres nebulosus* ), along with *Rhinecanthus aculeatus* and *Pomacanthus semicirculatus* , showed no significant difference in shrimp consumption across treatments. These species consumed most of the shrimps in all three treatments. Foraging efficiency also showed a high variation among three species of chaetodontids. Neither *C. auripes* nor *C. lunula* exhibited significant differences in foraging efficiency, regardless of the presence or absence of *Galaxaura* (Wilcoxon signed‐rank tests: *p* > 0.05), consuming approximately 40%–60% of the shrimp in all treatments. However, only *C. vagabundus* showed a significantly decreased foraging efficiency in both *Galaxaura* treatments (Wilcoxon signed‐rank tests: *p* = 0.02). In behavior, unlike herbivorous fish, ten nonherbivorous fish species showed no significant differences in feeding frequency between the *Galaxaura* and plastic mimic treatments, indicating that they were equally able to forage in both treatments (Figure 4b).

## 4 Discussion

### 4.1 Response of Hard Corals

Our results indicated that *Galaxaura divaricata* significantly suppressed hard corals and induced coral bleaching, highlighting strong nonphysical allelopathic effects linked to its presence in preliminary tests. The areas of bleaching observed in two hard coral species were significantly larger in the *Galaxaura* treatments than in the control and plastic mimic treatments. Similar findings have been reported in other studies (Del Monaco et al. 2017; Rasher and Hay 2010), suggesting that chemical metabolites produced by allelopathic macroalgae are the primary cause of coral bleaching, rather than physical contact or shading. Overall, we demonstrate that *Galaxaura* exerts strong allelopathic influences.

### 4.2 Response of Herbivorous Fishes

We found that most herbivorous fish significantly decreased their foraging efficiency and avoided contacting *Dictyota* when *Galaxaura* occurred. Red algae species from Nemaliales (e.g., Galaxauraceae and Liagoraceae) are known to be unpalatable to various herbivores, and their presence can indirectly shield neighboring algae from grazing pressure (Doropoulos et al. 2014, 2017; Loffler et al. 2015; Roff, Doropoulos, et al. 2015). We found that even a small piece of *Galaxaura* thallus could potentially influence the foraging behaviors of most herbivorous fishes (unpublished data). Moreover, our results also showed that ten herbivorous fishes did not consume 100% of the *Dictyota* biomass even in plastic mimic treatments. We observed that some herbivorous fishes attempted to bite forcefully into the algae but were unable to reach deeper parts of both *Galaxaura* thallus or the plastic mimic. These observations suggest that tough branched structures may serve as physical barriers from herbivores, and become a partial refuge for small‐sized epiphytes. Since the presence of *Galaxaura* can negatively affect the grazing efficiency of herbivorous fishes, it may form stable shelters and recruitment sources for many algae in reef systems. This may explain why rich epiphytes are often observed growing on or nearby the thalli of *Galaxaura divaricata* in the Dongsha Atoll (Nieder et al. 2019, 2022).

Herbivorous fishes from different trophic guilds showed varying degrees of decline in feeding quantity when facing *Galaxaura*, suggesting species‐specific differences in tolerance. The foraging behaviors of herbivorous fishes may not respond consistently to *Galaxaura* and its associated epiphytes. Four acanthurids and *Kyphosus* are typical fish browsers and can remove large amounts of biomass of fleshy algae as they forage across reefs (Hoey and Bellwood 2009; Johansson et al. 2013; Streit et al. 2015). We found that these taxa consistently avoided any treatment with *Galaxaura*, indicating that the chemicals produced by this allelopathic alga strongly repel these browsers. Our results also suggest that during macroalgal blooms, the trophic role of browsers in coral reefs may be highly suppressed by chemically rich allelopathic macroalgae. However, some herbivorous fish species showed a certain degree of tolerance to *Galaxaura*. Our results also indicated that siganids and scarids retained the ability to contact and consume macroalgae associated with *Galaxaura*. Studies from the Great Barrier Reef (Fox et al. 2009; Loffler et al. 2015) and Fiji (Rasher and Hay 2010) showed that siganids are capable of feeding on chemically rich algae, such as *Galaxaura* and the filamentous toxic green alga: *Chlorodesmis fastigiata* . These observations suggest that siganids are more tolerant of chemically rich macroalgae than acanthurids and *Kyphosus*. In addition, three species of Scaridae were also able to consume parts of *Dictyota* in the *Galaxaura* treatments. In particular, *Calotomus carolinus* exhibited the highest foraging efficiency among ten herbivorous fishes in the *Galaxaura* treatments. We even observed direct bite marks—despite only small pieces of thallus—on *Galaxaura* made by *C. carolinus* . The specialized beaks of scarids may help them break down the calcified thalli of *Galaxaura* and consume its thallus and epiphytes on it. These results indicate that siganids and scarids can remain effective herbivore functional groups even in reefs heavily dominated by allelopathic macroalgae. Our results highlight the importance of herbivore diversity in coral reef ecosystems, which plays a critical role in algal control in coral reef ecosystems (Streit et al. 2015; Vergés et al. 2012). Different feeding guilds (e.g., browsers, scrappers, grazers) and species of herbivores exhibit varying levels of resistance to allelopathic chemicals, as well as distinct preferences and feeding strategies, which may be key to suppressing macroalgal outbreaks and maintaining coral reef resilience.

### 4.3 Response of Nonherbivorous Fishes

Our study offers the first quantitative results on the foraging efficiency and behavior of nonherbivorous fishes when facing allelopathic macroalgae outbreaks. Macroalgae are considered critical habitats or shelters for small‐sized mobile invertebrates (Roff et al. 2013; Tano et al. 2016). Hay et al. (1989, 1990) showed that unpalatable macroalgae can be a refuge for small crustaceans in temperate regions. Despite the potential refuge function of allelopathic macroalgae, their effects on the foraging efficiency of nonherbivorous fishes have rarely been discussed. Although live crustaceans were not included in the experimental design, our results still suggest that the macroalgae effectively impeded fish foraging behavior. In this study, unlike ten herbivorous fish, ten benthic‐feeding carnivores generally showed no significant difference in both foraging efficiency and frequency between the *Galaxaura* and plastic mimic treatments, indicating that the presence of living *Galaxaura* thallus (with their chemicals) may not repel them. This suggests that these nonherbivorous fishes might use nonolfactory senses (e.g., sight or tactile sensation) for foraging in macroalgae thalli. From our perspective, *Galaxaura* likely interfered with their foraging behavior primarily through a simple physical barrier rather than chemical deterrence. Moreover, we found that different carnivore fish species exhibited varying foraging abilities when facing a physical barrier of *Galaxaura* or the plastic mimic. From our results, most *Epinephelus merra* , *Lutjanus fulviflamma*, and *Plectorhinchus chaetodonoides* failed to capture shrimp in both *Galaxaura* and plastic mimic treatments. They attempted to strike at the *Galaxaura* or plastic mimic treatments using their mouths or bodies in an effort to capture the shrimps, but mostly failed in our records. The wide mouths and short snouts of these three species may be physically blocked by the calcified thalli of *Galaxaura*, resulting in low foraging efficiency in both *Galaxaura* and plastic mimic treatments. In contrast, two labrid species, as well as *Rhinecanthus aculeatus* and *Pomacanthus semicirculatus* , consumed most of the shrimps across all the treatments. The thalli of *Galaxaura* did not appear to suppress their foraging ability. These species have relatively small mouths and/or long snouts, which may facilitate their ability to search for and feed on small invertebrates within the barriers of *Galaxaura* thalli. McClanahan et al. (1999) also showed that the presence of macroalgae exhibits no significant influence on the foraging and habitat preference of carnivorous fishes, such as some balistids and labrids in Kenya. We also observed these species generally shelter in coral reefs with abundant *Galaxaura* in the Dongsha Atoll, suggesting that high *Galaxaura* coverage does not negatively impact their foraging performance and habitat preference. Our findings may suggest that rather than being chemically repelled, the calcified thalli of *Galaxaura* may physically obstruct the foraging in some nonherbivorous fishes. The foraging efficiencies of these fishes in response to *Galaxaura* likely depend on their oral morphology and feeding strategies.

Coral‐feeding animals may also avoid contact with corals treated with allelopathic macroalgae (Brooker et al. 2017; Clements and Hay 2015; Venera‐Ponton et al. 2011). Previous study demonstrated that several coral‐feeding chaetodontids (Brooker et al. 2016) and obligate corallivorous fish: *Oxymonacanthus longirostris* (Brooker et al. 2017) would reduce their feeding behaviors or alter their habitat preference in the presence of allelopathic macroalgae. However, in our study, we found a distinct response of opportunistic corallivorous fishes to the *Galaxaura* treatments. Unlike obligate corallivores, opportunistic corallivorous fish can feed on small invertebrates in benthic habitats and may therefore also be affected by allelopathic macroalgae. Two opportunistic coral feeders, *Chaetodon auripes* and *C. lunula* , could consume parts of shrimps in both *Galaxaura* and plastic mimic treatments. Their longer snouts may allow them to penetrate barriers of *Galaxaura* or plastic mimic treatment and capture the invertebrate prey within. The foraging efficiencies and frequencies of these two chaetodontids were not significantly different between the *Galaxaura* and control treatments, indicating that the chemicals of *Galaxaura* may not repel them. In contrast, *C. vagabundus* exhibited a low foraging efficiency in *Galaxaura* treatments. Since the three chaetodontid species share similar long snout morphology, it remains unclear why such closely related species responded differently to the allelopathic macroalga. It is likely that different Chaetodontidae species also exhibited varying degrees of tolerance to the chemicals in the allelopathic macroalgae.

### 4.4 Ecosystem Implications

Our findings indicate that the ecological impacts of a shift toward dominance by allelopathic macroalgae may go beyond direct interactions with herbivorous fish. While herbivores are typically viewed as the main regulators of macroalgal abundance, our results show that allelopathic macroalgae can also influence the foraging behavior of nonherbivorous fish by changing the accessibility of prey and the structure of benthic habitats. These changes can reshape trophic interactions across various feeding groups and alter energy transfer within reef food webs, leading to downstream effects on fish assemblage structure, ecosystem services, and even fisheries productivity.

To isolate the effects of *Galaxaura* on fish foraging, we conducted controlled tank experiments that minimized environmental variability. However, the simplified nature of the tanks limits our ecological inferences. First, both sample sizes and the variation in body length were restricted, and the fish were tested individually. Body size is particularly important for herbivorous fish, as larger individuals generally exhibit greater forces, mobility, and a higher capacity to remove macroalgae (Adam et al. 2018; Munsterman et al. 2021). Additionally, schooling behavior may have influenced our results, as fish on natural reefs often forage in groups and within multispecies assemblages, where collective foraging can enhance feeding efficiency (Brandl and Bellwood 2014; Choat 1991; Hoey and Bellwood 2009).

Secondly, food availability was limited to *Dictyota* and shrimp meat, whereas natural *Galaxaura* thalli host a diverse array of epiphytes and invertebrates (Nieder et al. 2019). Variation in epiphyte preferences among herbivorous fish could affect foraging success, and the movement of invertebrates within the thallus network may further limit the foraging success of carnivorous fish. Finally, since we did not extract or manipulate allelopathic chemicals, we could not directly attribute the observed effects on other organisms to chemical toxicity. However, chemical cues may play a role, alongside structural factors, in the differences observed in feeding selectivity.

Despite these limitations, our study provides clear evidence that allelopathic macroalgae influence foraging behavior in both herbivorous and nonherbivorous fish. Future research should further investigate the toxic chemical compounds associated with various allelopathic macroalgae and evaluate their effects on benthic substrates. More importantly, in situ investigations and experiments are needed to examine the associated epiphytic and invertebrate communities and to validate these patterns under natural reef conditions following macroalgal phase shifts.

As macroalgal phase shifts become more common on degraded reefs, changes in foraging patterns across different trophic groups may lead to cascading effects on community composition, habitat use, and ecosystem processes, including fisheries productivity and the provision of ecosystem services. Therefore, understanding these indirect and community‐wide effects is crucial for evaluating reef resilience and predicting how ecosystems will function amid ongoing macroalgal proliferation.

## 5 Conclusions

Overall, this study provides a new insight into the potential impact of allelopathic macroalgal overgrowths on fish foraging behavior and trophic structures in coral reef ecosystems. For palatable algae and small benthic invertebrates, our results suggest that allelopathic macroalgae may serve as an ideal refuge from predators. We also found that the effects of allelopathic macroalgae on fish foraging efficiency were species‐specific. While some fish species continued to forage within the chemical‐rich allelopathic macroalga, others were strongly repelled. The overgrowth of allelopathic macroalgae reduced the foraging efficiency of most herbivores, particularly browser species, as well as large carnivores.

Reduced foraging efficiency may decrease fish preference for areas with abundant allelopathic macroalgae. This shift could lead to changes in fish species composition, habitat preferences, and a reduction in functional diversity within coral reef systems. When macroalgae overgrow, the efficiency of trophic transfer within reef systems may decline, as food sources and foraging grounds become limited by the presence of allelopathic macroalgae. In such circumstances, coral reef systems may struggle to recover, experiencing decreased resilience due to a lower abundance and functional capacity of both herbivores and carnivores. Understanding the interactions between allelopathic macroalgae and fish feeding guilds is essential for uncovering the mechanisms that underlie coral reef resilience, especially in degraded reef systems. Our findings suggest that maintaining functional diversity within fish feeding guilds and preserving specific herbivorous functions—such as those provided by siganids and scarids—is critical for enhancing coral reef resilience against phase shifts driven by macroalgal overgrowth. Furthermore, allelopathic macroalgae may change reef consumer assemblages and trophic pathways by altering community structure and the composition of functional guilds.

## Author Contributions

**Chen‐Lu Lee:** conceptualization (equal), data curation (equal), formal analysis (lead), investigation (lead), methodology (equal), validation (equal), visualization (lead), writing – original draft (lead), writing – review and editing (equal). **Pi‐Jen Liu:** conceptualization (equal), data curation (supporting), methodology (equal), project administration (equal), resources (equal), software (equal), supervision (equal), validation (equal). **Shao‐Lun Liu:** conceptualization (equal), funding acquisition (equal), methodology (equal), project administration (equal), resources (equal), supervision (equal), validation (equal). **Hsing‐Juh Lin:** conceptualization (equal), funding acquisition (equal), project administration (equal), resources (equal), software (equal), supervision (equal), validation (equal), writing – review and editing (equal).

## Funding

This research project was supported by the Ministry of Science and Technology of Taiwan (107‐2119‐M‐110‐009) and Marine National Park Headquarters, National Park Service, Ministry of the Interior. This work was financially supported in part by the “Innovation and Development Center of Sustainable Agriculture” from the Featured Areas Research Center Program within the Higher Education Sprout Project by the Ministry of Education (MOE) of Taiwan.

## Conflicts of Interest

The authors declare no conflicts of interest.

## Supporting information


**Data S1:** ece373106‐sup‐0001‐supinfo.xlsx.

## Data Availability

All the required data are uploaded as Supporting Information.
